# Supplementary material for: Modeling glioblastoma heterogeneity as a dynamic network of cell states
Source: Mol Syst Biol. 2021 Sep 16;17(9):e10105. doi: 10.15252/msb.202010105 (PMC8444284; doi:10.15252/msb.202010105)
Supplement: Supplementary file 6 — Source Data for Figure 5 [file MSB-17-e10105-s004.zip › Figure5A_sourcedata/GSEA_3017/nosigdb_stateA.GseaPreranked.1622795026485/NB_KI_CLUSTER5.html]

Details for gene set NB\_KI\_CLUSTER5[GSEA]

|  || Dataset | state53017 |
| Phenotype | NoPhenotypeAvailable |
| Upregulated in class | na\_pos |
| GeneSet | NB\_KI\_CLUSTER5 |
| Enrichment Score (ES) | 0.46019474 |
| Normalized Enrichment Score (NES) | 2.702789 |
| Nominal p-value | 0.0 |
| FDR q-value | 0.0 |
| FWER p-Value | 0.0 |
Table: GSEA Results Summary

  

Fig 1: Enrichment plot: NB\_KI\_CLUSTER5      
 Profile of the Running ES Score & Positions of GeneSet Members on the Rank Ordered List

  

| PROBE | GENE SYMBOL | GENE\_TITLE | RANK IN GENE LIST | RANK METRIC SCORE | RUNNING ES | CORE ENRICHMENT || 1 | CDK1 |  |  | 3 | 1.006 | 0.0536 | Yes |
| 2 | KIF20B |  |  | 10 | 0.749 | 0.0895 | Yes |
| 3 | BUB3 |  |  | 12 | 0.740 | 0.1302 | Yes |
| 4 | KPNA2 |  |  | 20 | 0.667 | 0.1604 | Yes |
| 5 | HIST1H4C |  |  | 21 | 0.659 | 0.1976 | Yes |
| 6 | PCLAF |  |  | 28 | 0.637 | 0.2272 | Yes |
| 7 | CKS2 |  |  | 40 | 0.587 | 0.2487 | Yes |
| 8 | CKS1B |  |  | 50 | 0.530 | 0.2691 | Yes |
| 9 | DBF4 |  |  | 55 | 0.514 | 0.2939 | Yes |
| 10 | MIS18BP1 |  |  | 64 | 0.495 | 0.3133 | Yes |
| 11 | SGO2 |  |  | 73 | 0.478 | 0.3319 | Yes |
| 12 | UBE2T |  |  | 83 | 0.470 | 0.3489 | Yes |
| 13 | DUT |  |  | 87 | 0.466 | 0.3720 | Yes |
| 14 | PLK1 |  |  | 89 | 0.464 | 0.3971 | Yes |
| 15 | TUBB4B |  |  | 93 | 0.462 | 0.4200 | Yes |
| 16 | ZWINT |  |  | 106 | 0.447 | 0.4325 | Yes |
| 17 | HIST2H2AC |  |  | 131 | 0.419 | 0.4307 | Yes |
| 18 | HELLS |  |  | 135 | 0.417 | 0.4511 | Yes |
| 19 | MCM7 |  |  | 171 | 0.394 | 0.4363 | Yes |
| 20 | TRIP13 |  |  | 209 | 0.370 | 0.4181 | Yes |
| 21 | SMC4 |  |  | 217 | 0.367 | 0.4313 | Yes |
| 22 | H2AFZ |  |  | 245 | 0.351 | 0.4226 | Yes |
| 23 | CLSPN |  |  | 274 | 0.336 | 0.4119 | Yes |
| 24 | PRC1 |  |  | 350 | 0.310 | 0.3500 | Yes |
| 25 | ANP32B |  |  | 357 | 0.307 | 0.3610 | Yes |
| 26 | ECT2 |  |  | 365 | 0.305 | 0.3708 | Yes |
| 27 | BRCA1 |  |  | 373 | 0.302 | 0.3804 | Yes |
| 28 | CKAP2 |  |  | 379 | 0.301 | 0.3921 | Yes |
| 29 | CENPW |  |  | 386 | 0.299 | 0.4026 | Yes |
| 30 | TEX30 |  |  | 388 | 0.298 | 0.4184 | Yes |
| 31 | RACGAP1 |  |  | 399 | 0.293 | 0.4243 | Yes |
| 32 | HMGB1 |  |  | 403 | 0.292 | 0.4376 | Yes |
| 33 | HMGB2 |  |  | 424 | 0.288 | 0.4327 | Yes |
| 34 | NCAPD2 |  |  | 440 | 0.283 | 0.4328 | Yes |
| 35 | ANP32E |  |  | 453 | 0.280 | 0.4359 | Yes |
| 36 | ATAD2 |  |  | 470 | 0.275 | 0.4345 | Yes |
| 37 | EZH2 |  |  | 471 | 0.275 | 0.4500 | Yes |
| 38 | SHCBP1 |  |  | 477 | 0.274 | 0.4602 | Yes |
| 39 | UBE2S |  |  | 507 | 0.269 | 0.4447 | No |
| 40 | CIP2A |  |  | 522 | 0.266 | 0.4449 | No |
| 41 | RANBP1 |  |  | 536 | 0.263 | 0.4460 | No |
| 42 | FBXO5 |  |  | 557 | 0.258 | 0.4394 | No |
| 43 | SPDL1 |  |  | 560 | 0.258 | 0.4519 | No |
Table: GSEA details [plain text format]

  

Fig 2: NB\_KI\_CLUSTER5: Random ES distribution      
 Gene set null distribution of ES for **NB\_KI\_CLUSTER5**
